# Optical biometry and influence of media opacity due to cataract on development of axial length in NorthEast Indian paediatric patients- A prospective study

**DOI:** 10.1186/s12886-021-02138-4

**Published:** 2021-10-22

**Authors:** Harsha Bhattacharjee, Suklengmung Buragohain, Henal Javeri, Saurabh Deshmukh

**Affiliations:** Sri Sankaradeva Nethralaya, 96 Basistha Road, Saurabh Nagar, Beltola Tiniali, Guwahati, Assam 781028 India

**Keywords:** Media opacity, Congenital cataract, Axial length, Genetics of ocular growth

## Abstract

**Aim:**

To study the influence of media opacity due to cataract on the development of axial length in paediatric patients from North-East India, using optical biometry.

**Method:**

This is a prospective, observational study, including consecutive patients attending the paediatric ophthalmology clinic, over a period of 1 year. Patients with other ocular and systemic diseases, unfit for optical biometry measurements due to dense cataract, nystagmus and strabismus were excluded and rest divided into three groups after proper age matching – 1. Group A (Bilateral cataract) 2. Group B (Unilateral cataract) 3. Group C (Bilateral normal). The axial length of the various groups was analysed using independent sample test (for bilateral cataract group) and paired t-test (for unilateral cataract group). Linear regression analysis between age and axial length was done.

**Results:**

A total of 177 patients were included.80 cases in Group A (bilateral cataract), 18 cases in Group B (unilateral cataract) and 79 in Group C (bilateral normal) The mean age of the patients in all the groups was 8.88 ± 3.51 years (range: 1–17 years). The bivariate analysis and simple linear regression revealed a statistically significant correlation between age and AL in case of cataractous eyes. (Pearson’s coefficient: 0.341, *p* < 0.001). The mean AL was significantly longer (*p* = 0.013) in the cataractous eyes (mean = 23.38 ± 2.08 mm) of Group A(bilateral cataract) in the 7–12 years age group as compared to the bilaterally normal eyes (mean AL = 22.57 ± 0.70 mm) of patients in the same age group in Group C. The mean AL of cataractous eyes in group B (unilateral cataract) (mean = 22.46 ± 1.73 mm) as compared to the fellow normal eyes, (mean = 21.87 ± 0.97 mm) was not statistically significant.

**Conclusion:**

Cataractous eyes have an abnormal axial length development. The influence of media opacity due to cataract on development of axial length in paediatric eyes in the North-East Indian population is variable, in line with global data on the same. Although there is some influence of media opacity, the exact nature is not clearly understood and may have a crucial interaction with genetic and other environmental factors. Genetic testing integrated with biometric analysis is recommended for further understanding of the ocular growth and development.

## Introduction

Paediatric cataract accounts for 7.4–15.3% of cases of childhood blindness [1, 2]. Khanna et al. found amblyopia in 50.9% cases of congenital and developmental cataract and amblyopia was more common in congenital than developmental cataract (93% vs 14.9%, *p* < 0.001) [3].

Uncertainty, regarding selection of appropriate intra-ocular lens (IOL) power is a very important limitation [4, 5] that seriously compromises the visual outcome. The unpredictable development of post-surgical axial growth in children probably triggered by the media opacity at the time of development may contribute to poor visual outcome.

Various hypothesis have been postulated on the probable factors regulating the growth of the axial length (AL) of the eye though the exact nature is yet to be explained. The current understanding is based on the observations from avian [6] and primate models [7]. Two school of thoughts exist on what plays a major role in determination of AL. One group believe it to be majorly guided by the media opacity and another believe it to be due to genetic factors [8].

The effect of the media opacity on the AL of the growing human eye has been studied by the various authors [9–12]. A majority of these studies have used ultrasound biometry for calculation of AL. In the present study, we have studied the influence of media opacity due to cataract on the development of axial length in paediatric eyes in a north-east Indian population, with the help of optical biometry (Carl Zeiss Meditech IOL Master), for a more accurate measurement of AL. The effects of media opacity on the AL of the eye, has been studied and the findings compared with current literature, so as to determine the predominant factor regulating eye growth. This knowledge can assist in more reliable prediction of AL and IOL power calculation for a more satisfactory visual outcome in paediatric cataract patients.

## Materials and methods

This was a prospective, observational study that was conducted after obtaining approval from Sri Sankaradeva Nethralaya Institutional Ethics Committee and it adhered to the Declaration of Helsinki. All consecutive cases, over a 1 year period, attending the paediatric outpatient department (OPD) of the institute underwent a comprehensive ocular and systemic evaluation. Out of these, all cases having central cortical, zonular or central lenticular opacity and in which the disc and retina were hazily visible on direct ophthalmoscopic examination, with the other eye normal, were labelled as unilateral cataract. An emmetropic eye without any media opacity was considered as a normal eye. Bilateral cataract was labelled when both eyes had a zonular, central cortical or central lenticular opacity providing a hazy view of the disc and retina. Subjects who were not willing to participate, those with any other ocular disease, history of trauma, ocular surgery or any systemic diseases were excluded. Situations wherein optical biometry was not possible such as cases with dense cataract, nystagmus, strabismus or patients uncooperative or unable to fixate for optical biometry measurements were excluded from the study. Informed consent was taken in all cases from the parents/guardians of the patients. Patients up to the age of 17 years were included in the study. One hundred seventy seven patients who fulfilled the inclusion criteria were further divided into the following three groups: Group A – Bilateral cataract, Group B – Unilateral cataract with normal fellow eye and Group C – Bilateral normal eyes. Eighty eyes out of 158 eyes in group A and 79 eyes in group C, each, were selected by systematic sampling method.

The selected eyes in group A and C each, were further divided into 3 age groups-1-6 years, 7–12 years and 13–17 years for age-based analysis.

In all patients, non-contact biometry, working in the principle of partial coherence interferometry (Carl Zeiss Meditech IOL Master) was used to measure the AL. ?(the distance from the corneal vertex to the retinal pigment epithelium). Optical measurements were taken before any ocular examination that required eye contact or application of drops, was done. The machine was regularly calibrated against the ultra-high resolution 40Mhz Grieshaber system which is an internal algorithm to maintain the accuracy of measurement within ±0.02 mm or better. The instrument table, the head rest, and the IOL Master were adjusted before each measurement, to ensure that the child was seated in a relaxed and stable position. The measurement procedure was explained and the requirement for the patient’s head to remain in a fixed position with no unnecessary eye movements was also pointed out. Before taking the measurements, the patients were asked to blink and then to focus on the fixation light. It was also reaffirmed whether the fixation light was visible to the patient. The AL, keratometry readings and predicted IOL power calculation was measured for both the eyes in each subject. For each eye, five biometric measurements were acquired and average of these five readings were considered for the study.

The biometric data was collected and entered into Microsoft Excel 2013 (Redmond, WA, USA). Descriptive statistical methods, together with 95% confidence interval (CI), were used to assess the characteristics such as AL and age. Independent samples t-test was used for analysis of AL between the bilateral cataractous and bilateral normal eyes. For unilateral cataracts, a paired *t*-test was used to compare AL of the cataractous eye with the non-cataractous fellow eye. Pearson correlation between attributes with scatter plot displaying regression line was depicted. IBM SPSS 20 and JMP 10 of SAS 9.3 was used in data processing.

## Results

A total of 177 patients were involved in the study.80 cases in Group A (bilateral cataract), 18 cases in Group B (unilateral cataract) and 79 in Group C (no cataracts) (Table 1). The mean age in Group A was 8.13 ± 3.32 years (range: 1–16 years). The mean age in Group B was 7.33 ± 3.71 years (range: 2–17 years). The mean age in Group C was 9.98 ± 3.35 years (range: 3–16 years). The mean age of the patients in all the groups was 8.88 ± 3.51 years (range: 1–17 years).

**Table 1:** Group-wise distribution of Patients

	Group	No. of eyes
A	**Bilateral Cataract**	80
	1–6 years	24
	7–12 years	49
	13–17 years	07
B	**Normal + Cataract**	18+ 18
C	**Bilateral Normal (Control Group)**	79
	1–6 years	16
	7–12 years	47
	13–17 years	07
	**Total**	177

Mean Axial length in Group A was 22.95 ± 2.26 mm. The mean axial length of the cataractous eyes in Group B was 22.46 ± 1.73 mm and that of the fellow normal eyes was 21.87 ± 0.97 mm. In Group C, mean axial length was of the cataractous eyes was 22.54 ± 0.84 mm (Table 2).

**Table 2:** Showing Mean Axial Length (AL) and Standard Deviation (SD) values given Eye-wise for each group

	Group		AL
A	Bilateral Cataract	Mean	22.66
		SD	3.24
		n	80
B	Normal + Cataract	Mean (of cataractous eye)	22.46
		SD	1.73
		n	18
C	Bilateral Control	Mean	22.54
		SD	0.84
		n	79

### Comparison between age and axial length (Fig. 1)

Simple linear regression analysis with age as an independent variable demonstrated a positive significant effect of age on increase in axial length in cataractous eyes in the study, as demonstrated by the equation below.

$$AL=21.12+0.22\ast age,{R}^2=0.116,P<0.01$$

**Fig. 1:**
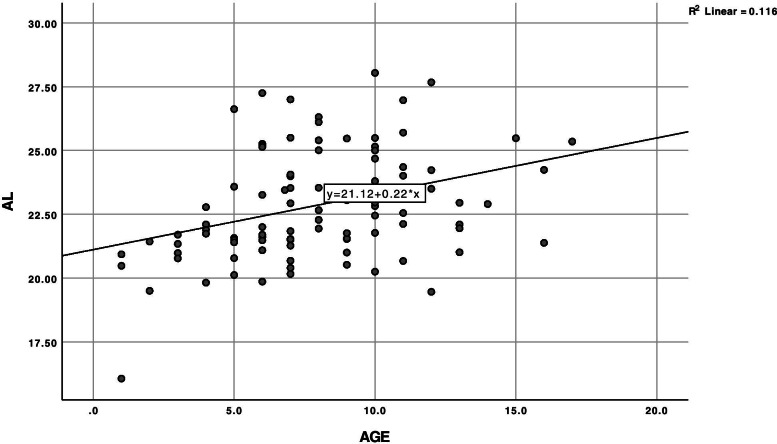
Bivariate Scatter Plot of Axial length (AL) (mm) and Age (Years). The scatter plot demonstrates the positive correlation of axial length with age in cataractous eyes. y = AL; x: age

Scatter plot of axial length and age showed a statistically significant relationship,with a 0.22 mm increase in axial length per year. (Pearson’s correlation co-efficient = 0.341, *p* < 0.001). (Fig. 1).

### Comparison between the Cataractous eyes of group a and Normal eyes of group C

#### Age group 0–6 years (Table 3)

The difference in the AL between the cataractous eye of Group A (22.10 ± 2.60 mm, *n* = 24) and normal eyes of Group C (*n* = 16, mean AL = 22.34 ± 0.84 mm) was not statistically significant (*P* = 0.72).

**Table 3:** Comparing AL differences between Group A (Bilateral Cataract) and Group C (Bilateral Control). Ages 1–6 (Years)

Group	n	Mean	SD	Mean Difference	SE Difference	*p*-value
Group A	24	22.10	2.26	−0.25	0.69	0.72 ^NS^
Group C	16	22.35	1.07			

^NS^ Not Significant *p* > .05, *SE* Standard Error

#### Age group 7–12 years: (Table 4)

The difference in the AL between the cataractous eye of Group A (23.38 ± 2.08 mm, *n* = 49) and normal eyes of Group C (*n* = 47, mean AL = 22.57 ± 0.70 mm) was statistically significant (*P* = 0.013).

**Table 4:** Comparing AL differences between Group A (Bilateral Cataract) and Group C (Bilateral Control). Ages 7–12 (Years)

Group	n	Mean	SD	Mean Difference	SE Difference	*p*-value
Group A	49	23.38	2.07	0.81	0.32	0.01^S^
Group C	47	22.57	0.70			

^S^ Significant *p* > .05, *SE* Standard Error

#### Age group 13–17 years (Table 5)

The difference in the AL between the cataractous eye of Group A (22.87 ± 1.58 mm, *n* = 07) and normal eyes of Group C (*n* = 16, mean AL = 22.64 ± 1.00 mm) was not statistically significant (*P* = 0.68).

**Table 5:** Comparing AL differences between Group A (Bilateral Cataract) and Group C (Bilateral Control). Ages 13–17 (Years)

Group	n	Mean	SD	Mean Difference	SE Difference	*p*-value
Group A	07	22.87	1.58	0.23	0.54	0.68^NS^
Group C	16	22.64	1.00			

^NS^ Significant *p* > .05, *SE* Standard Error

### Unilateral versus Normal fellow eyes in unilateral cataract group (Table 6)

The difference in the AL between the cataractous eyes (mean AL = 22.46 ± 1.73; *n* = 18) and the fellow eyes (mean AL = 21.87 ± 0.97 mm; n = 18) was not statistically significant (*P* = 0.80).

**Table 6:** Comparing difference in AL between Cataractous and Normal Eyes of each patient in Group B

Eye	Mean	SD	Mean Difference	SE mean	*p*-value
Cataractous eyes (n = 18)	22.46	1.73	0.59	1.35	0.08^NS^
Non- Cataract (n = 18)	21.87	0.97			

^NS^ Not Significant *p* > 0.05; *SE* Standard Error

### Comparison between mean AL of unilateral versus bilateral Cataractous eyes

The difference between the mean AL of Group A (22.95 ± 2.26 mm, *n* = 80) and Group B cataractous eyes (22.46 ± 1.73; n = 18) was not statistically significant. (*p* = 0.39).

## Discussion

Numerous factors determine how the axial growth of the eye behaves in paediatric eyes. Some of the factors which have been known to be associated are age, general health and growth, clarity of ocular media. Even more factors seem to come into play in cases of paediatric cataracts, such as child’s age at surgery, aphakia, pseudophakia, cataract laterality, and visual deprivation [13–15].

The theories explaining the regulation of ocular growth are diverse. The key premise amongst them is that ocular growth has mainly two components-the active component, governed by the retinal image formation and the passive component, controlled by genetic factors [8]. How exactly they control the axial growth, and which component plays a major role is yet unanswered.

The role of retinal image formation has been widely studied in animal as well as human subjects. The concept is that the degradation in quality of retinal image (form deprivation) or the alteration in focal point of image (lens defocus) provides a feedback for adjustment of AL and mechanical modelling of the eye [16]. Media opacity compromises the image quality on the retina and results in form deprivation [17]. Large alteration of retinal image quality due to congenital cataract [17, 18], corneal opacity [9, 19], vitreous haemorrhage [20], ptosis [21], and other ocular diseases [22] might influence the growth pattern of the paediatric eye. Regarding lens defocus, a number of studies have been conducted in animals as well as humans. In avian experiments, increased AL has been noted after myopic defocus [6]. In animal models, Smith III et al. found a decrease in AL in 5 out of 8 monkeys with hyperopic defocus [7]. In humans, Nickla et al. found variable change in the AL with hyperopic defocus depending on the time of the day [23]. Read et al. demonstrated that a short hyperopic defocus resulted in an increase in the AL and in a study conducted by Chakraborty et al., the eye undergoing myopic defocus, developed increase in AL [16, 24].

Axial length is expected to increase with age. Our study, too, found a significantly positive correlation between age and axial length in cataractous eyes,(*p* < 0.01),with an increase in 0.22 mm axial length for every 1 year of age. The cataractous eyes seem abnormal to begin with, and appear to not follow the expected amount of progression with age, as is reported in case of normal eyes [25]. Consecutive biometric measurements of each case would provide the exact relationship and progression of development with age and can be explored in further studies. Our study also demonstrates that the mean AL was significantly longer (*p* = 0.013) in the cataractous eyes (mean = 23.38 ± 2.08 mm) of Group A in the 7–12 years age group as compared to the bilaterally normal eyes (mean AL = 22.57 ± 0.70 mm) of patients in the same age group in Group C. In the age groups 1–6 years and 13–17 years, respectively, there was no statistically significant difference in the axial lengths between Group A and group C patients. The process of emmetropisation is most active around the age of 8 years [8] and that may possibly indicate as to why there was a significant difference in the AL values among group A and group C eyes in the age group of 7–12 years. The difference could signify the deviation occurring in the process of emmetropisation in the cataractous eyes owing to either media opacity or genetic factors.

The cataractous eyes in group B (mean = 22.46 ± 1.73 mm) had a longer mean axial length as compared to the fellow normal eyes, (mean = 21.87 ± 0.97 mm) although it was again, not statistically significant.

A lesser amount of retinal form deprivation is expected in a case of unilateral cataract, and a resultant reduced feedback for AL growth as compared to bilateral cataractous eyes. A larger mean AL was noted in the bilateral cataractous group (22.95 ± 2.26 mm, *n* = 80) in our study, in comparison to the mean AL in the unilateral cataractous group (mean AL = 22.46 ± 0.97), but was not statistically significant (*p* = 0.07), again questioning the influence of media opacity.

Other reports that have compared the AL of the cataractous eye against the AL of the fellow normal eye have also found variable results. Capozzi et al. (USG biometry) found a shorter AL in cases of bilateral congenital cataract and no change in cases of unilateral congenital cataracts [26]. Trivedi et al. (Biometry method not mentioned) reported longer AL in case of bilateral congenital cataract than those with unilateral cataract in paediatric patients younger than 60 months of age and a shorter AL when compared to unilateral cataract paediatric patients older than 60 months of age [27]. In a study by Lambert et al., cases of unilateral congenital cataract had a shorter AL in comparison to the fellow eye [28].

In the previously mentioned studies, Capozzi et al. utilised an ultrasonographic biometer whereas the biometry method was not mentioned by Trivedi et al. and Lambert et al. Other studies comparing the biometry characteristics in paediatric cataract cases have also employed the ultrasonic biometer for axial length measurement [1, 7, 8, 26, 29]. This technique being a contact procedure, can give falsely shorter axial length readings, especially in pediatric patients because of lower corneal and scleral rigidity [16, 30]. The procurement of inaccurate measurements is also higher due to lack of cooperation in paediatric patients [30]. Optical biometry technique, employed in our study, has been shown to provide contact-free measurements, observer independence and high reproducibility and accuracy over conventional ultrasound [31].

The fluctuating nature of the above findings points towards another school of thought regarding the growth of the eyeball- the vast universe of genetics. Physiologically, the eye at birth is hypermetropic; the AL grows as the child ages so as to reach an emmetropic state. Emmetropization harmonizes the globe elongation, with the optical power of the cornea and lens to reduce refractive error [32]. The genetic regulation of refraction may be via the control of the process of emmetropisation, to result in proportionate growth of the various components of refraction [33].

AL loci using gene-based tests have been identified and suggest that the growth of different parts of the eyeball are regulated by different gene expression. Some genes such as ZC3H11A, GJD2, and LAMA2 show constant changes in expression in different eye sections in the same direction while other genes like RSPO1, C3orf26, and ZNRF3 show variable directions of differential expression. Compensatory changes in corneal curvature or optical power with axial growth in order to balance their effect on spherical equivalent, maybe regulated by genes such as PSPO1 [34–37]. Apart from the studies on specific genes related to the axial growth of the eyeball, strong evidence of genetic influence on the axial length has also been demonstrated by studies in twins [38, 39]. Dirani et al. also showed high heritability for AL in which 90% of the variance was accounted to be due to additive and dominant genetic effects [40].

The results of our study, along with the review of the various studies which have been done previously, show conflicting results regarding the influence of media opacity on AL, suggesting that media opacity has some influence overall, the exact nature of which is not clearly understood and may have a crucial interaction with genetic and other environmental factors.

To the best of our knowledge, this study is a first of its kind from the North-Eastern part of the country. Future studies incorporating both, refractive and genetic testing of children with cataract are required. The duration and entity of the visual deprivation and amblyopia may influence biometric parameters, and keeping in mind a tendency for delayed presentation of pediatric cataracts to a tertiary institution, a thorough effort was made to establish the age of onset in every case, although objective records were not available. Quantification of the amblyopia and its influence on the axial length was beyond the scope of this study. Only patients with visually significant media opacity in whom optical biometry readings were possible were included in the study, adding to its limitation.

In conclusion, results of our study on the influence of media opacity amongst the pediatric cataractous eyes of North East India, re-iterate the variable, somewhat transient and subordinate influence of media opacity on the development of AL, consistent with multiple studies reported from different parts of the world; and calls for in-depth studies involving genetic analysis for exploring the interaction of genetic factors in the proportionate development of AL and the various refractory components, which might help us understand this process better in the future.

## Data Availability

Data pertaining to the results and conclusions of this study are included within the manuscript file. Any additional data can be obtained by writing to the corresponding author for the same. Details of the corresponding author are as below: Dr. Henal Javeri, Sri Sankaradeva Nethralaya, 96 Basistha Road, Saurabh Nagar, Beltola Tiniali, Guwahati, Assam-781028 Email: hjjaveri20@gmail.com Phone No: +919821404406.

## References

[1] Khokhar SK, Pillay G, Dhull C (2017). Pediatric cataract. Indian J Ophthalmol.

[2] Sheeladevi S, Lawrenson JG, Fielder AR (2016). Global prevalence of childhood cataract: a systematic review. Eye (Lond).

[3] Khanna RC, Foster A, Krishnaiah S (2013). Visual outcomes of bilateral congenital and developmental cataracts in young children in South India and causes of poor outcome. Indian J Ophthalmol.

[4] Sinskey RM, Amin PA, Lingua R (1994). Cataract extraction and intraocular lens implantation in an infant with a monocular congenital cataract. J Cataract Refract Surg.

[5] Vasavada A, Chauhan H (1994). Intraocular lens implantation in infants with congenital cataracts. J Cataract Refract Surg.

[6] Wallman J, Adams JI (1987). Developmental aspects of experimental myopia in chicks. Vis Res.

[7] Smith EL, Hung L, Harwerth RS (1994). Effects of optically induced blur on the refractive status of young monkeys. Vis Res.

[8] Brown NP, Koretz JF, Bron AJ (1999). The development and maintenance of emmetropia. Eye (Lond).

[9] Gee SS, Tabbara KF (1988). Increase in ocular axial length in patients with corneal opacification. Ophthalmology.

[10] Calossi A (1994). Increase of ocular axial length in infantile traumatic cataract. Optom Vis Sci.

[11] Kun L, Szigeti A, Bausz M (2018). Preoperative biometry data of eyes with unilateral congenital cataract. J Cataract Refract Surg.

[12] Prado R, Silva V, Schellini A (2016). Congenital and developmental cataract: axial length and keratometry study in Brazilian children. Arq Bras Oftalmol.

[13] Wang D, Ding X, Liu B (2011). Longitudinal changes of axial length and height are associated and concomitant in children. Invest Ophthalmol Vis Sci.

[14] Ojaimi E, Morgan IG, Robaei D (2005). Effect of stature and other anthropometric parameters on eye size and refraction in a population-based study of Australian children. Invest Ophthalmol Vis Sci.

[15] Vasavada AR, Raj SM, Nihalani B (2004). Rate of axial growth after congenital cataract surgery. Am J Ophthalmol.

[16] Chakraborty R, Read SA, Vincent SJ, Ang M, Wong T (2020). Understanding myopia: pathogenesis and mechanisms. Updates on myopia.

[17] Von Noorden GK, Lewis RA (1987). Ocular axial length in unilateral congenital cataracts and blepharoptosis. Invest Ophthalmol Vis Sci.

[18] Rabin J, Van Sluyters RC, Malach R (1981). Emmetropization: a vision-dependent phenomenon. Invest Ophthalmol Vis Sci.

[19] Meyer C, Muller MF, Duncker GW (1999). Experimental animal myopic models are applicable to human juvenile-onset myopia. Suru Ophthalmol.

[20] Miller-Meeks MJ, Bennett SR, Keech RU (1990). Myopia induced by vitreous haemorrhage. Am J Ophthalmol.

[21] Hoyt CS, Stone RD, Former C (1981). Monocular axial myopia associated with neonatal eyelid closure in human infants. Am J Ophthalmol.

[22] Nathan J, Kiely PM, Crewther SG (1985). Disease-associated visual image degradation and spherical refractive error in children. Am J Optom Physiol Optic.

[23] Nickla D, Jordan K, Yang J (2017). Brief hyperopic defocus or form deprivation have varying effects on eye growth and ocular rhythms depending on the time-of-day of exposure. Exp Eye Res.

[24] Read SA, Collins MJ, Sander BP (2010). Human optical axial length and defocus. Invest Ophthalmol Vis Sci.

[25] Larsen JS (1971). The sagittal growth of the eye. IV. Ultrasonic measurement of the axial length of the eye from birth to puberty. Acta Ophthalmol.

[26] Capozzi P, Morini C, Piga S (2008). Corneal curvature and axial length values in children with congenital/infantile cataract in the first 42 months of life. Invest Ophthalmol Vis Sci.

[27] Trivedi RH, Wilson ME (2007). Biometry data from caucasian and african-american cataractous pediatric eyes invest. Ophthalmol Vis Sci.

[28] Lambert SR, Buckley EG, Drews-Botsch C (2010). The infant aphakia treatment study: design and clinical measures at enrollment. Arch Ophthalmol.

[29] Sahu S, Panjiyar P (2019). Biometry characteristics in congenital cataract patients before surgery in a tertiary eye care Centre in Nepal. Saudi J Ophthalmol.

[30] Gursoy H, Sahin A, Basmak H (2011). Lenstar versus ultrasound for ocular biometry in a pediatric population. Optom Vis Sci.

[31] Wilson ME, Trivedi RH (2012). Axial length measurement techniques in pediatric eyes with cataract. Saudi J Ophthalmol.

[32] Wallman J, Winawer J (2004). Homeostasis of eye growth and the question of myopia. Neuron..

[33] Norton TT, Siegwart JT (1995). Animal models of emmetropization: matching axial length to the focal plane. J Am Optom Assoc.

[34] Cheng CY, Schache M, Ikram MK (2013). Nine loci for ocular axial length identified through genome-wide association studies, including shared loci with refractive error. Am J Hum Genet.

[35] Dirani M, Shekar SN, Baird PN (2008). Evidence of shared genes in refraction and axial length: the genes in myopia (GEM) twin study. Invest Ophthalmol Vis Sci.

[36] Guggenheim JA, Zhou X, Evans DM (2013). Coordinated genetic scaling of the human eye: shared determination of axial eye length and corneal curvature. Invest Ophthalmol Vis Sci.

[37] Guggenheim JA, McMahon G, Kemp JP (2013). Genomewide association study for corneal curvature identifies the platelet-derived growth factor receptor alpha gene as a quantitative trait locus for eye size in white Europeans. Mol Vis.

[38] He M, Hur YM, Zhang J (2008). Shared genetic determinant of axial length, anterior chamber depth, and angle opening distance: the Guangzhou twin eye study. Invest Ophthalmol Vis Sci.

[39] Lopes MC, Andrew T, Carbonaro F (2009). Estimating heritability and shared environmental effects for refractive error in twin and family studies. Invest Ophthalmol Vis Sci.

[40] Dirani M, Chamberlain M, Shekar SN (2006). Heritability of refractive error and ocular biometrics: the genes in myopia (GEM) twin study. Invest Ophthalmol Vis Sci.

